# *Crataegus oxyacantha* Extract as a Biostimulant to Enhance Tolerance to Salinity in Tomato Plants

**DOI:** 10.3390/plants11101283

**Published:** 2022-05-11

**Authors:** Imane Naboulsi, Reda Ben Mrid, Abdelhamid Ennoury, Zakia Zouaoui, Mohamed Nhiri, Widad Ben Bakrim, Abdelaziz Yasri, Aziz Aboulmouhajir

**Affiliations:** 1 Organic Synthesis, Extraction and Valorization Laboratory, Faculty of Sciences Ain Chock, Hassan II University, Km 8 El Jadida Road, Casablanca 20000, Morocco; imane.naboulsi@um6p.ma (I.N.); aziz.aboulmouhajir@etu.univh2c.ma (A.A.); 2 AgroBioSciences Program, Mohammed VI Polytechnic University (UM6P), Lot 660, Hay Moulay Rachid, Ben Guerir 43150, Morocco; widad.benbakrim@um6p.ma (W.B.B.); abdelaziz.yasri@inra.ma (A.Y.); 3 Laboratory of Biochemistry and Molecular Genetics, Faculty of Sciences and Technologies of Tangier, BP 416, Tangier 90000, Morocco; abdelhamid.ennoury@etu.uae.ac.ma (A.E.); zakia.zouaoui@etu.uae.ac.ma (Z.Z.); mnhiri@uae.ac.ma (M.N.); 4 African Sustainable Agriculture Research Institute (ASARI), Mohammed VI Polytechnic University (UM6P), Laâyoune 70000, Morocco; 5 The National Institute of Agronomic Research (INRA), Av. Annasr, Rabat 10000, Morocco

**Keywords:** *Solanum lycopersicum*, biostimulant, antioxidant enzymes, *Crataegus oxyacantha* extract, salt stress

## Abstract

Salinity is a severe abiotic problem that has harmful impacts on agriculture. Recently, biostimulants were defined as bioprotectant materials that promote plant growth and improve productivity under various stress conditions. In this study, we investigated the effect of *Crataegus oxyacantha* extract as a biostimulant on tomato plants (*Solanum lycopersicum*) grown under salt stress. Concentrations of 20 mg/L, 30 mg/L, and 70 mg/L of *C. oxyacantha* extract were applied to tomato plants that were grown under salt stress. The results indicated that plants that were treated with *C. oxyacantha* extract had a higher ability to tolerate salt stress, as demonstrated by a significant (*p* < 0.05) increase in plant growth and photosynthetic pigment contents, in addition to a significant increase in tomato soluble sugars and amino acids compared to the control plants. In the stressed tomato plants, malondialdehyde increased and then decreased significantly with the different concentrations of *C. oxyacantha* extract. Furthermore, there was a significant improvement in the antioxidant enzyme activities of superoxide dismutase (SOD), glutathione peroxidase (GPx), glutathione S-transferase (GST), and glutathione reductase (GR) in the stressed plants, especially after treatment with 70 mg/L of the extract. Overall, our results suggest that *C. oxyacantha* extract could be a promising biostimulant for treating tomato plants under salinity stress.

## 1. Introduction

Abiotic stress limits plant growth and productivity worldwide. Excessive salinity and drought are the most significant environmental stresses that lead to crop loss [1]. Soil salinity affects agricultural productivity in many parts of the world. In fact, salt stress belongs to the category of the most destructive stresses leading to a reduction in the yield of major crops in several countries [2]. Globally, over 833 million hectares are affected by soil salinity, accounting for 8.7% of the total land area. This value may grow significantly in the coming years as soil salinity can be exacerbated by climate change and unsustainable agricultural production practices [3].

Salinity affects seed germination and plant growth because of the excess uptake of toxic substances. Seeds and seedlings are particularly vulnerable to increased salinity. It has been found that salinity induces photorespiration and reactive oxygen species (ROS), such as superoxide radicals (O_2−_) and hydrogen peroxide (H_2_O_2_), which cause damage to membranes, photosynthetic pigment, protein, lipids, and metabolic pathways [4]. Therefore, to overcome the effects of salt stress, plants need several tolerance mechanisms to improve growth parameters, nutrient uptake, and photosynthetic and antioxidant defense system activation. To deal with the damage caused by ROS, plants have evolved different defense strategies by producing non-enzymatic and enzymatic antioxidants, which protect the plant tissues from the oxidative damage [5].

Tomato (*Solanum lycopersicum* L.) is an important crop that is grown within a wide range of production systems. Tomato plants are listed in the top of 20 commodities that are grown under salt stress worldwide. In terms of production, tomato is considered a worldwide crop and according to the FAOSTAT (2020), the planted area of tomato exceeds 4.7 million ha, which produces a yield that exceeds 182 million tons [6]. Tomato is moderately salt-tolerant but remains susceptible to cell damage from high salinity. Previous studies have reported that tomato plant growth, metabolism, and yield are affected by osmotic and ionic stress caused by salinity [7,8].

Plant-based biostimulants are rich in bioactive compounds. These natural compounds have been reported to enhance different physiological processes in plants that may improve nutrient use efficiency and, therefore, increase plant growth and development. Interest in the use of these biostimulants has increased over recent decades, both in terms of improving plant growth and protecting plants from abiotic stresses [9,10]. The biostimulant candidate that was used in this study was an aqueous extract of *Crataegus oxyacantha*, a plant that contains several secondary metabolites in different areas (fruit, leaves, and flowers) and has been used for the treatment of various pharmacological activities, including antiarrhythmic activity, cardiotonic activity, and hypolipidemic activity [11,12,13]. Many studies have shown that the use of biostimulants can alleviate salt stress in plants. Therefore, this study aimed to investigate the effects of different concentrations of *Crataegus oxyacantha* extract (COE) on alleviating the adverse effects of salinity in tomato plants. *Crataegus oxyacantha* extract was chosen because it contains a large number of flavonoids (predominantly flavonols and flavones) (ref), phenolcarboxylic acids (mainly chlorogenic acid) [12], and oligomeric proanthocyanidin compounds, which may play an important role in alleviating oxidative stress [11]. The biostimulant effect of the extract was evaluated by measuring physiological and biochemical parameters, such as growth parameters, chlorophyll, soluble sugar, and amino acid content, and also some antioxidant enzyme activities (SOD, GR, GPx, GST, PEPc, and GDH).

## 2. Results and Discussion

### 2.1. Effects of Crataegus oxyacantha Extract on Physiological Parameters of Tomato Plants under Salt Stress

Salinity stress is considered as the most severe abiotic stresses, which results in considerable losses in crop productivity. In general, the physiological parameters of plants are the first visual indication that provide a global idea of the interactions between the environment and the growing conditions of plants [14].

The 12 tomato plants exposed to salt stress in this study showed a significant decrease in their physiological parameters. Salt stress exposure significantly decreased plant fresh weight and height by 37.9% and 18.41%, respectively, compared to the control plants (Table 1).

Concerning the *C. oxyacantha* extract treatments, irrigation with 20 mg/L resulted in a 110% increase in fresh weight, while 30 and 70 mg/L insignificantly decreased fresh weight by 9.75% and 44.4%, respectively, compared to the control plants (Table 1). Furthermore, treatment with 20, 30, and 70 mg/L of *C. oxyacantha* extract increased plant height by 0.82%, 1.37%, and 17%, respectively, compared to the plants treated with NaCl only (Table 1). Our results showed that the tomato plants that were exposed to salt stress had significantly decreased shoot length and fresh weight compared to the control plants. These results agreed with those obtained by Alzahib et al. (2021) [15], in which a high concentration of NaCl (0.3 M) decreased tomato plant growth, leaf area, plant height, and fresh and dry root weights.

The application of *C. oxyacantha* extract treatment to tomato plants under salt stress has been shown to neutralize the NaCl effect by increasing the physiological parameters of the plants (length and weight). Our results agreed with previous studies by Ceccarini et al. (2019) [16], which suggested the beneficial effects of the use of polyphenol-enriched extracts as plant biostimulants on corn plant growth parameters, such as height and fresh weight. The authors reported that spelt husk extracts contain three hydroxycinnamic acids (p-coumaric, caffeic, and ferulic acids) in addition to two simple phenolic compounds (thep-hydroxybenzoic and syringic acids) that may lead to a significant increase in shoot growth and height compared to plants treated with NaCl only. Another study that was led by Ozfidan-Konakci et al. (2015) [17] showed that gallic acids can induce effective protection against salt stress in rice seedlings.

Mrid et al. (2021) [18] also mentioned that phenolic acids have been widely studied for their ability to alleviate abiotic stresses, especially drought and salinity stresses. Moreover, carrot (*Daucus carota*) root extracts possess various bioactive compounds that could improve yield and plant growth parameters under salinity stress [19]. Benazzouk et al. (2020) [20] examined the effects of vermicompost as a biostimulant on tomato salt stress resistance. The authors established that vermicompost decreases the impact of salinity on leaf senescence.

### 2.2. Effects of Cataegus oxyacantha Extract on Photosynthetic Pigment (Chlorophyll a, Chlorophyll b, and Total Chlorophyll) Enzymes in Tomato Seedlings under Salt Stress

Chlorophyll is the principal pigment that is found in most oxygenic photosynthetic organisms. Chlorophyll content in plants is the main factor that reflects their photosynthetic rate [21]. Many studies have demonstrated that variations in pigment content can provide valuable insight into the physiological performance of leaves and indicate their photosynthesis ability and overall plant health condition (stress or diseases) [22].

**Figure 1:**
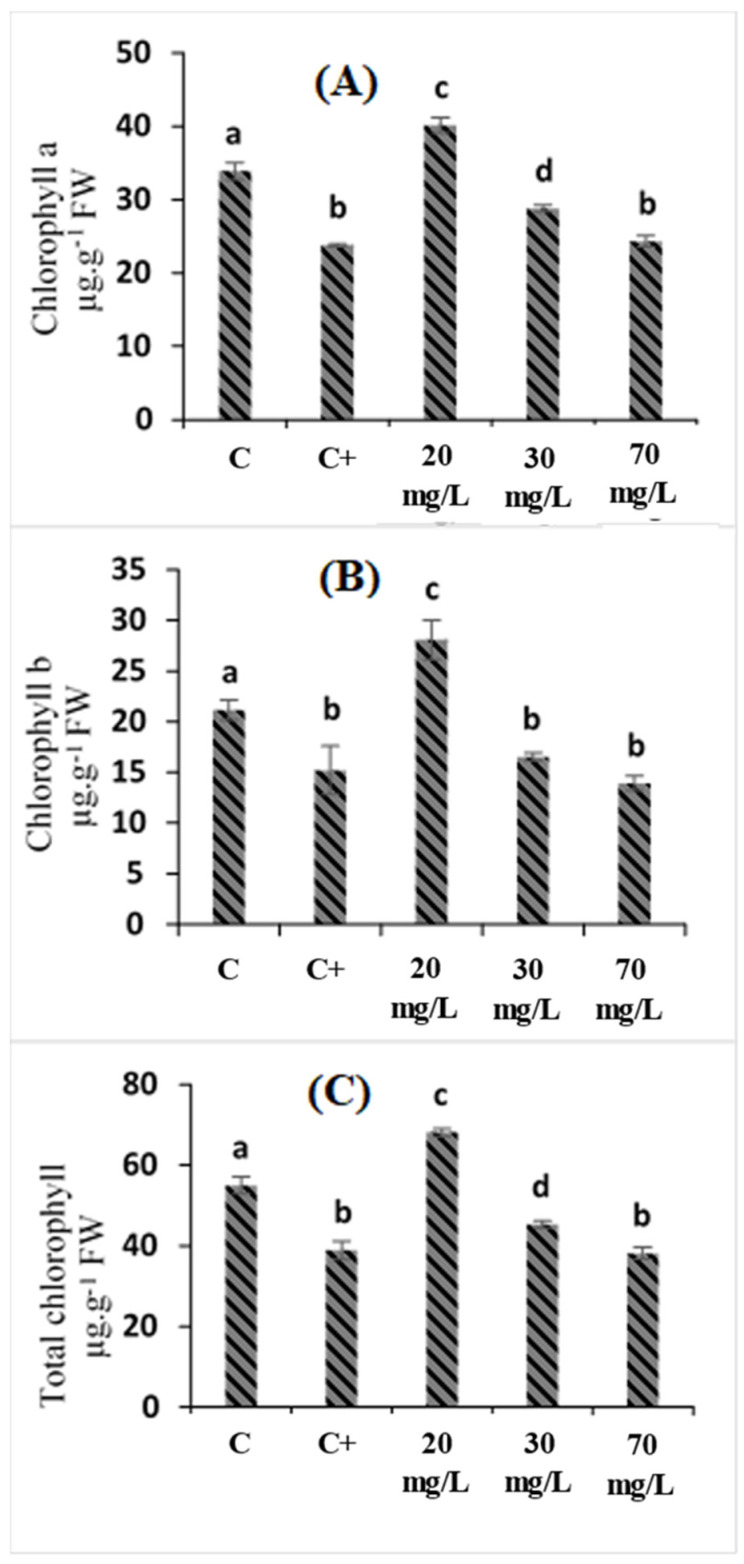
The effects of *Crataegus oxyacantha* extract on pigment chlorophyll content in tomato plants under salt stress: (**A**) chlorophyll a; (**B**) chlorophyll b; (**C**) total chlorophyll. Different letters indicate significant differences between conditions (*p* < 0.05) within conditions according to Tukey’s multiple range test.

Our results demonstrated that chlorophyll a and b content decreased significantly in the leaves of tomato plants that were treated with 75 mM of NaCl (Figure 1). The chlorophyll a content presented the significant reduction of 29.75% compared to the control plants (Figure 1A), while chlorophyll b content was reduced by 28.22% under the same conditions (Figure 1B).

The treatment of stressed plants with 20 mg/L of *C. oxyacantha* extract significantly reduced the stress effect on chlorophyll a and b content, while treatment with 30 and 70 mg/L had a non-significant effect on chlorophyll pigments.

The treatment with 20 mg/L of *C.* *oxyacantha* extract significantly enhanced the chlorophyll a content in untreated and treated plants from 23.77 µg·g^−1^ FW to 40.13 µg·g^−1^ FW, respectively (Figure 1A). Under the same conditions, the chlorophyll b content also increased to reach the higher value of 28.07 µg·g^−1^ FW in comparison to untreated plants (15.18 µg·g^−1^ FW) (Figure 1B).

Our results showed that 75 mM of NaCl significantly decreased the total chlorophyll, chlorophyll a, and chlorophyll b contents. Moreover, 20 mg/L of *C. oxyacantha* extract improved the pigment content of the stressed plants. The application of the extract on tomato seedlings significantly enhanced their concentrations of photosynthesis pigments compared to those of the control plants (under salt stress).

Our results agreed with those of Bacha et al. (2017) [23], in which a decrease in the chlorophyll content of *Solanum lycopersicum* that was subjected to salinity stress was observed. The same results were also found by Latique et al. (2021) [24]. The authors investigated the effect of salt stress on wheat (*Triticum Durum L.*) and the ability of *Ulva rigida* water extract to improve salinity tolerance in wheat (*Triticum Durum L.*).

The study by Kabiri et al. (2018) [25] showed that the foliar application of melatonin increased the chlorophyll content in *Dracocephalum moldavica* under abiotic stress. Mrid et al. (2021) [18] also mentioned that the application of caffeic acids has been reported to improve biomass and chlorophyll accumulation and reverse the negative impacts of salt stress.

The increase in chlorophyll content may be related to some of the bioactive compounds that have been identified in *C. oxyacantha* extract, such as glycinebetaine [26]. This component has been reported as being able to inhibit chlorophyll degradation, thus leading to a delay in the loss of photosynthetic activity during storage conditions in isolated chloroplasts [24]. Moreover, *C. oxyacantha* extract is rich in antioxidant molecules, which positively correlate with improvements in the production of photosynthetic pigments in wheat plants that are grown under salinity stress.

### 2.3. Effects of C. oxyacantha Extract on Malondialdehyde (MDA), Soluble Sugar, and Amino Acid Content in Tomato Seedlings under Salt Stress

To investigate the effects of crude *C. oxyacantha* extract, different biochemical parameters were analyzed in this study, including malondialdehyde MDA, soluble sugar, and amino acids.

In general, MDA, which is a cytotoxic product of membrane lipid peroxidation, has been considered as an indicator of oxidative damage under drought, cold shock, and salt stress conditions [27]. However, soluble sugars and free amino acids have been proven to serve as osmoprotectants in various plant species [28].

**Figure 2:**
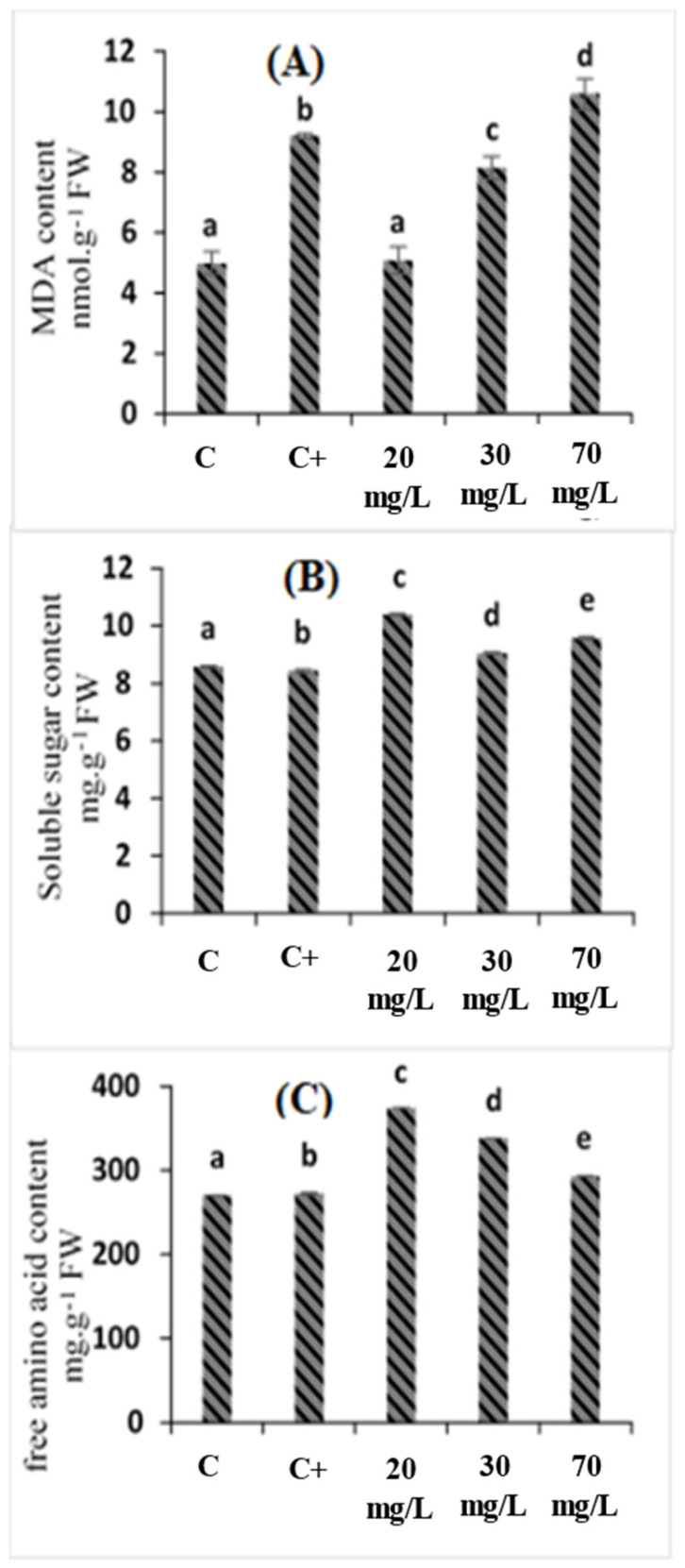
The effects of *C. oxyacantha* extract on malondialdehyde (MDA), soluble sugar, and free amino acid content in tomato plants under salt stress: (**A**) MDA content; (**B**) soluble sugar content; (**C**) free amino acid content. Different letters indicate significant differences between conditions (*p* < 0.05) within conditions according to Tukey’s multiple range test.

From the obtained results, salt treatment intensely increased the MDA content in leaves by 84.69% compared to the control plants (Figure 2A). Treatment with 20 and 30 mg/L of *C. oxyacantha* extract effectively reversed these effects (Figure 2A), especially 20 mg/L, with which the MDA content of leaves decreased by 44.78% compared to the salt-treated plants. These findings suggested that the appropriate concentration *C. oxyacantha* extract significantly decreased the lipid peroxidation of tomato seedlings under salt stress. This oxidative marker indicates that salinity has altered membrane functionality. These findings were in line with previous studies on tomato plants, which described the oxidative stress that is induced by salinity [29,30], and other plant species [31,32,33].

As shown in Figure 2B,C, the salt treatment induced an increase of 0.76% in the free amino acid content in tomato leaves under salt stress compared to the control plants. In contrast, the soluble sugar content decreased by 1.77% compared to the salt-treated plants. The treatment of stressed plants with 20, 30, and 70 mg/L of *C. oxyacantha* extract significantly increased the free amino acid and soluble sugar content, especially 20 mg/L, with which those contents increased by 37.47% and 23.16%, respectively, compared to control (salt-treated plants). The increase in total free amino acid content under salt stress was in accordance with the study of Noman et al. (2018) [34], who demonstrated that free amino acids accumulate in *Triticum monococcum* L. under salt stress. Our results also showed that 20 mg/L of *C. oxyacantha* extract treatment stimulated the synthesis of soluble sugar content. These results followed Mrid et al. (2021) [18], who mentioned that soluble sugar content increases after spraying the aerial parts of *Mentha arvensis* with calliterpenone, which is extracted from *Callicarpa macrophylla*, at a concentration of 0.01 mM, twice a day for up to 45 days. Another study that was led by Rosa et al. (2009) [35] demonstrated that soluble sugar is considered to be a physiological index of salt stress tolerance. Carbohydrates, as with soluble sugar, provide energy and solutes for osmoprotectants.

### 2.4. Effects of C. oxyacantha Extract on Antioxidant Enzymes in Tomato Plants under Salt Stress

Several studies have demonstrated the relation between salt stress and antioxidant systems in plants. In general, the tolerance of tomato plants to salinity is related to the increase in antioxidant enzyme activities (superoxide dismutase (SOD), ascorbate peroxidase (APX), and catalase (CAT)) as a strategy to counter the oxidative stress caused by ROS [18,36].

**Figure 3:**
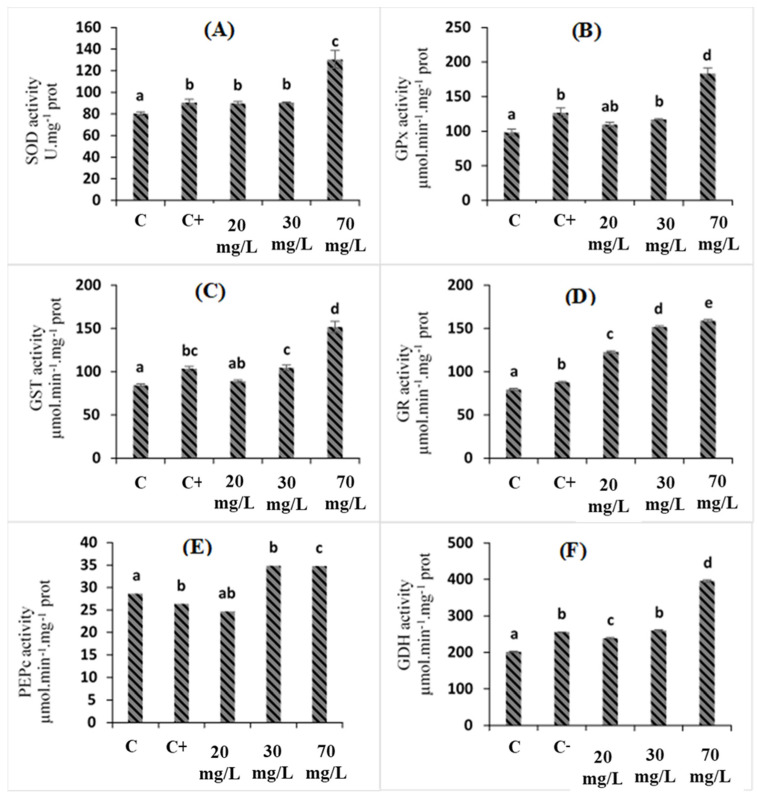
The effects of *C. oxyacantha* extract on enzyme systems in tomato plants under salt stress. Abbreviations: (**A**) SOD, superoxide dismutase; (**B**) GPx, glutathione peroxidase; (**C**) GST, glutathione S-transferase; (**D**) GR, glutathione reductase; (**E**) PEPc, phosphoenolpyruvate carboxylase; (**F**) GDH, glutamate dehydrogenase. Different letters indicate significant differences between conditions (*p* < 0.05) within conditions according to Tukey’s multiple range test.

The present study demonstrated that the enzymes SOD, glutathione peroxidase (GPx), glutathione S-transferase (GST), and glutathione reductase (GR) increased significantly in leaves under salt stress by 12.5%, 22.65%, 22.44%, and 10.49%, respectively, compared to the control plants (Figure 3). These activities were upregulated in *C*. *oxyacantha* extract-treated plants, especially with 70 mg/L treatment, with which SOD, GPx, GST, and GR activities increased by 44.25%, 44.78%, 46.7%, and 99.87%, respectively, compared to the salt-treated plants (Figure 3A–F).

In response to stress, plants set up different mechanisms for non-enzymatic and enzymatic antioxidants to eliminate the generated ROS. Out of the enzymatic antioxidants, SOD is considered to be the first barrier against ROS [37]. This enzyme catalyzes the dismutation of superoxide anion radical (O_2−_) into hydrogen peroxide (H_2_O_2_) [38]. The subsequent H_2_O_2_ is then neutralized by its conversion into H_2_O through a reaction that is catalyzed by glutathione peroxidase (GPx). In addition to these activities, it is known that GST could reduce a wide range of organic hydroperoxides in the presence of GSH [39]. Moreover, GST enzymes can provide protection to plants that are grown under salinity stress by eliminating the effects of this stress on lipid peroxidation [40] while GR permits the production of large amounts of GSH, which is considered as an essential element for the metabolic pathways that are associated both with biomass production and ensuring protection under stress conditions [20].

Our study showed that the application of *C. oxyacantha* extract as a biostimulant enhanced antioxidant activities. The same result was obtained by Latique et al. (2021) [24]. The authors claimed that *Ulva rigida* extract enhances the antioxidant enzyme activities of wheat under salinity stress. Another study that was led by Li et al. (2013) [41] showed that pretreatment with ferulic acids increases SOD activity in *Cucumis sativus* seedlings.

In addition to the antioxidant enzymes, salt stress also affects carbon–nitrogen assimilation activities. From the obtained results, the GDH and PEPc activities presented different aspects under salt stress than those presented by the control. The phosphoenolpyruvate carboxylase activity (PEPc) in the leaf extracts of tomato plants that were subjected to 75 mM of NaCl is shown in (Figure 3E,F). Phosphoenolpyruvate was inhibited by 8.06% in the stressed tomato plants compared to the control plants, while an upregulation in GDH activity of 27.1% compared to the control was also observed.

The three concentrations of *C. oxyacantha* extract stimulated PEPc and GDH activities. The high values of these enzyme activities were present in the case of treatment with 70 mg/L of *C. oxyacantha* extract. PEPc activity increased from 26.34 µmol/min/mg (plants under salt stress) to reach 34.75 µmol/min/mg (plants treated with 70 mg/L of *C. oxyacantha* extract), while GDH activity increased from 255.60 µmol/min/mg (plants under salt stress) to 395.93 µmol/min/mg (plants treated with 70 mg/L of *C. oxyacantha* extract). The upregulation of PEPc could be related to salt stress tolerance.

Liu et al. (2021) [42] demonstrated that the overexpression of PEPc activity significantly increases salt tolerance in transgenic tobacco plants. In general, this enhancement is related to proline production, which plays an important role in protecting membrane integrity, photosynthesis, regulating osmotic balance, and activating the ROS scavenging system. In fact, GDH could possess a predominant role in ammonium assimilation and glutamate biosynthesis, which lead to an increased glutamate pool for proline synthesis, as suggested by Kaur et al. (2018) [43].

Furthermore, the upregulation of enzyme activities that was induced by *C. oxyacantha* extract treatment could be related to the biocompounds that are present in the plant extract, such as soluble sugar, polyphenol, and proteins. These compounds could stimulate antioxidant enzymes [44].

## 3. Material and Methods

### 3.1. C. oxyacantha Collection and Preparation of Extracts

*Crataegus oxyacantha* is a medicinal plant of the Rosaceae family, which is known as hawthorn. it is native to Europe and the plant is widely distributed. The plant is present in different parts of the Mediterranean coast of North Africa [45]. The plant is a source of several chemical compounds in different parts, such as flavonoids that can vary from 0.1% to 1% in its fruit and from 1% to 2% in its leaves and flowers, depending on climatic and environmental conditions, as well as oligomeric proanthocyanidins that play an important role in the pharmacological sector, such as improving antioxidant activity, according to several scientific articles [11,12]. For our experiments, the mature fruit of *C. oxyacantha* was collected from the region of Taza (latitude: 34°12′36″ N and longitude: 4°00′35″ W), Morocco, which has a Mediterranean climate.

In total, 4.5 g of mature *C. oxyacantha* fruit was extracted in 45 mL of H_2_O under continuous shaking (250 rpm) at room temperature. The extract was filtered through Whatman filter paper and centrifuged at 6000× *g* for 10 min. Then, the supernatant was evaporated in an incubator at 40 °C. The dried extract was used to prepare different concentrations of the plant extract (*C. Oxyacantha*), which were then kept at 4 °C until use.

### 3.2. Plant Material and Bioassays for Tomato Growth and Treatments

*Solanum lycopersicum* L. plants were purchased from Commercial Seeds for Farm, Greenhouse, and Garden Growing. The tomato seeds were sterilized using 5% of NaOCl for 15 min and were then rinsed with sterile water. The seeds were cultivated in a starter tray that contained soil. One seed of *S. lycopersicum* was planted per cell. After 1 week, the plants were subdivided into five classes:

1. Plants only treated with water (control);
2. Plants treated with 75 mM of NaCl;
3. Plants treated with 75 mM of NaCl + 20 mg/L COE (irrigation);
4. Plants treated with 75 mM of NaCl + 30 mg/L COE (irrigation);
5. Plants treated with 75 mM of NaCl + 70 mg/L COE (irrigation).

The 12 plants in each class were grown under controlled conditions at 28 °C day/21–22 °C night and a photoperiod of 12/12 h (light/dark). The plants were harvested after 4 weeks. The weights and lengths of the plants were measured and the leaves were stored at −80 °C until use.

### 3.3. Determination of Chlorophyll Content

Fresh leaf samples were placed in a mixture of 80% acetone in the dark and were incubated for 72 h. After chlorophyll extraction, the chlorophyll content was determined in three independent replicates using Arnon methods [46]. The absorbance was measured at 645 nm and 663 nm, and chlorophyll a, chlorophyll b, and total chlorophyll contents were estimated using the following formulae:

Chlorophyll a (mg L^−1^) = 12.7 × O.D._663_ − 2.69 × O.D._645_

Chlorophyll b (mg L^−1^) = 22.9 × O.D._645_ − 4.68 × O.D._663_

Chlorophyll _total_ (mg L^−1^) = Chlorophyll a + Chlorophyll b

where O.D._645_ and O.D._663_ are the optical densities at 645 and 663 nm, respectively.

### 3.4. Determination of MDA Content

The lipid peroxidation index malondialdehyde (MDA) was measured according to the method described for leaf senescence, which correlated with increased levels of membrane permeability and lipid peroxidation and decreased levels of superoxide dismutase and catalase with slight modifications. Plant cell homogenate was mixed with 4 mL of trichloroacetic acid (20%) and 0.67% thiobarbituric acid. After mixing with a vortex, the mixture was heated to 100 °C for 1 h. Then, *n*-butanol was added after the mixture had been cooled in ice. Then, the mixture was centrifugated at 1200× *g* for 12 min. Next, the supernatant was collected and the absorbance was measured at 532 and 600 nm. The unspecific activity was corrected by A_600_ − A_532._ The concentration of MDA was calculated using an extinction coefficient of 155 mM^−1^ cm^−1^.

### 3.5. Determination of Soluble Sugar and Amino Acid Content

The fresh leaf samples were extracted with 80% ethanol at 4 °C and were then centrifuged for 10 min at 10,000× *g*. Soluble sugars were determined according to the method of Yemm and Willis AJ (1954) [47]. A calibration curve was created using glucose to calculate the data as mg/g dry weight.

The amino acid content in the leaves was measured according to the method of Smith et al. (2015) [48]. Fresh leaves were ground in cold 80% ethanol. After 10 min, an aliquot of 500 µL of the extract was added to 1.5 mL of a 2% ninhydrin solution (which was solubilized in 0.2 M of citrate buffer, pH 5, and ethylene glycol). Then, the mixture was incubated at 100 °C for 15 min. Once cooled, 1.5 mL of ethanol (60%) was added and then an incubation of 1 h in the dark took place. Finally, the optical density at 546 nm was measured and the amino acid content was determined from the calibration curve that was produced using different concentrations of glycine.

### 3.6. Enzyme Extraction and Assays

For the enzyme extracts and assays, 0.2 g of fresh shoots were extracted in a mixture of 0.1 M of HEPES KOH, 20 µM of FAD, 10 mM of MgCl2, 1 mM of PMSF, and 14 mM of β-mercaptoethanol. The homogenate was centrifuged at 20,000× *g* for 20 min at 4 °C. Then, the supernatant was collected to determine the enzyme activities.

The activity of superoxide dismutase (SOD) was assayed by measuring its ability to inhibit the phytochemical reduction of nitro blue tetrazolium (NBT), as described by Bouchmaa et al. (2018) [49].

The activity of glutathione reductase (GR) was measured by following the change in absorption at 340 nm due to NADPH oxidation, as described by Rao et al. (1996) [50]. The product concentration was calculated using an extinction coefficient of 6.2 mM^−1^ cm^−1^. The assay of glutathione peroxidase (GPx) was measured by the spectrophotometric method of Bouchmaa et al. (2018) [49]. The measurement of the glutathione S-transferase (GST) activity was carried out using the method described by Latique et al. (2021) [24]. The reaction was measured at 340 nm and the concentration of products was calculated using a coefficient extinction of 9.6 mM^−1^ cm^−1^. The glutamate dehydrogenase (GDH) activity was measured according to Ben Mrid et al. (2018) [51]. The kinetic activity was determined spectrophotometrically by monitoring NADH at 340 nm.

To assess the phosphoenolpyruvate carboxylase (PEPc) enzymatic assay, ammonium sulfate was added to a volume of the supernatant to precipitate the enzymes of PEPc. The measurement of the PEPc activity was carried out following the method of Omari et al. (2016) [39]. The activity was assayed by spectrophotometrically monitoring NADH oxidation at 340 nm. One unit of PEPc was the amount of enzyme extract that was used to catalyze the transformation of 1 μmol of substrate per minute at 30 °C.

### 3.7. Statistical Analysis

Data were analyzed using the SPSS 25 package for Windows, v. 10.0.1. Additionally, ANOVA one factor and the Student–Newman–Keuls post-hoc test were used to compare the differences between the means (*p* < 0.05). Different letters indicated significant differences.

## 4. Conclusions

In conclusion, the results obtained from this study showed that the use of NaCl to stimulate salt stress induced many changes in tomato plants. A reduction in growth parameters, such as height, weight, and chlorophyll content, were observed. However, the stress also increased lipid peroxidation and stimulated antioxidant enzyme activities. Our study also indicated that the application of *C. oxyacantha* extract to tomato plants enhanced the plants’ tolerance to NaCl. Our use of the extract increased plant growth and photosynthetic pigment content. The extract also improved the activity of several antioxidant enzymes in the tomato plants, especially those treated with 70 mg/L of the extract. It can be concluded that *C. oxyacantha* extract may represent an effective tool to stimulate plant growth and alleviate the negative effects of salinity, which could improve the quality of plants and the quality of future fruit. Thus, further research is required to understand the molecular mechanisms for restoring the ion balance and alleviating the adverse effects of salinity.

## Figures and Tables

**Table 1:** The effects of *C. oxyacantha* extract on the traits of tomato seedlings affected by salt stress. Different letters indicate significant differences between conditions (*p* < 0.05) within conditions according to Tukey’s multiple range test.

	Control (Water)	Control + 75 mM NaCl	20 mg/L + 75 mM NaCl	30 mg/L + 75 mM NaCl	70 mg/L + 75 mM NaCl
**Plant Fresh Weight (mg)**	882 ± 48 ^a^	547 ± 35 ^b^	1153 ± 210 ^a^	493 ± 55 ^b^	304 ± 27 ^b^
**Plant Height (cm)**	17.8 ± 1.4 ^a^	14.5 ± 1.9 ^b^	14.7 ± 1.7 ^bc^	14.7 ± 0.5 ^c^	17 ± 1.3 ^a^

## Data Availability

The data presented in this study are available in insert article.
